# SOCIAL WELL-BEING OF OLDER PEOPLE IN ENGLAND SINCE THE PANDEMIC: DID ALREADY-ISOLATED OLDER ADULTS FARE WORSE?

**DOI:** 10.1093/geroni/igad104.1378

**Published:** 2023-12-21

**Authors:** Claryn Kung

**Affiliations:** University College London, London, England, United Kingdom

## Abstract

The COVID-19 pandemic and actions to reduce transmission (including stay-at-home orders and workplace closures) have had an impact on actual and perceived social isolation and support across all age groups. There was a clear increase in social isolation during the pandemic, especially among older adults, potentially due to the heightened risks of complications and mortality if infected with the novel coronavirus. In England, employing data from the nationally representative English Longitudinal Study of Ageing and using pre- (2018/19) and during pandemic data (June/July 2020 and November/December 2020), we found that overall, older adults who were already experiencing social isolation prior to the pandemic (isolated group) experienced a larger rise in loneliness and drop in social contact during the pandemic than older adults who were previously socially connected (non-isolated group). Results are robust even when we account for the fact that respondents socially isolated (including those not married and with infrequent contact with relatives and friends) felt lonelier and less supported even before the pandemic, and when we adjust for demographic and socioeconomic characteristics. Expanding on this work with post-pandemic data collected between October 2021 and March 2023, this paper describes loneliness, social contact, and social support pre-, during, and post-COVID-19 pandemic and investigates whether and to what extent trends in social well-being continued to diverge between the isolated and non-isolated groups.

